# Systems Librari-anship: A Practical Guide

**DOI:** 10.5195/jmla.2022.1358

**Published:** 2022-01-01

**Authors:** B. Lynn Eades

**Affiliations:** 1 beades@med.unc.edu, Technology Integration Librarian, University of North Carolina at Chapel Hill, Chapel Hill, NC

If the COVID-19 pandemic taught librarians anything, it is that having reliable catalogs, websites, guides, and electronic resources is crucial to continuing their mission of service to their patrons. While the move to electronic resources has been growing, so has the reliance on these systems and the ones who oversee them.

Brighid Gonzales has written a guide for those interested in becoming or transitioning into systems librarian-ship. As part of the Practical Guides for Librarians series, *Systems Librarianship: A Practical Guide* is an engaging overview of the complexities and variety of responsibilities a systems librarian may oversee. Sharing her personal experience of coming into a systems librarian position with no formal training in library school, she also shares the experiences of 200 system librarians who participated in a survey she conducted. Several in-depth interviews with some of the respondents end each chapter, further highlighting these experiences.

*System Librarianship: A Practical Guide* complements and updates the standard text for those new to systems librarianship, *The Accidental Systems Librarian* [1]. As in the *Accidental Systems Librarian,* Gonzales covers integrated library and content management systems, technology planning, and systems migration while expanding on web redesign strategies and emerging technologies such as augmented/virtual reality and drones. She highlights the need to build relationships with IT professionals both in libraries and in the academic or community setting, noting that for many the system librarian job is usually combined with other, more traditional library positions. Having those technical relationships can be a lifesaver when a problem arises.

At 168 pages, Gonzales offers an overview to an ever-expanding and changing area of librarianship. As more content moves online, the role of the systems librarian becomes even more essential. The references, in-depth interviews, and resources in this book give any new librarian or one moving into a systems role the help they need to be effective in their position.
